# Radiographic comparison of density and height of posterior maxillary bone after open sinus lift surgery with and without PRF

**DOI:** 10.15171/japid.2018.008

**Published:** 2018-12-25

**Authors:** Mohamad Taghi Chitsazi, Ali Hosien Dehghani, Amir Reza Babaloo, Sohrab Amini, Hadi Kokabi

**Affiliations:** ^1^Department of Periodontics, Dental School, Tabriz University of Medical Sciences, Tabriz, Iran; ^2^Department of Periodontics, Dental School, Islamic Azad University of Tabriz, Tabriz, Iran; ^3^Department of Periodontics, Dental School, Ardabil University of Medical Sciences, Ardabil, Iran; ^4^Department of Periodontics, Dental School, Hamedan University of Medical Sciences, Hamedan, Iran

**Keywords:** Dental implans, Platelet-Rich Fibrin, Sinus Floor Augmentation

## Abstract

**Background:**

Expansion of maxillary sinus towards the alveolar crest due to tooth loss or horizontal‒vertical resorption of the alveolar bone decreases the available bone for the placement of dental implants in the posterior maxilla. The method suggested for placing implants with a standard length is the use of sinus lift surgery with autogenous bone graft or bone substitute materials. The aim of the present research, with split-mouth design, was radiographic comparison of the density and height of the posterior of maxillary bone after open sinus lift procedure with and without PRF.

**Materials and Methods.:**

In this split-mouth clinical trial, 14 patients were evaluated, with complete or partial bilateral edentulism of the upper jaw. In each case, for the sinus lift surgery of the test side, PRF was used, while in the sinus lift surgery of the other side of the same patient no graft materials were used. After six months and before the second surgery, CBCT was used to evaluate bone density and height.

**Results:**

All the 41 implants were osseointegrated and were clinically stable. The bone height was 1.42 mm higher in the PRF group than the group without PRF, which was statistically significant. The mean density of the bone formed around the dental implants in the PRF group was 52.85 units higher than that of the group without PRF, which was statistically significant.

**Conclusion:**

Using PRF in sinus lift surgery might enhance the quantity and quality of bone formation.

## Introduction

Pneumatization of maxillary sinus towards the alveolar crest due to tooth loss or horizontal‒vertical resorption of the alveolar bone decreases the available bone to place dental implants in the posterior maxilla.^1,2^ The method proposed for placing implant with a standard length is the use of sinus lift surgery with autogenous bone graft or bone substitute materials. Recent advances in surgical techniques and graft materials have resulted in improved prognosis of implant treatment in the posterior maxillary region.^1,3^ Also, due to the high success rate, it can be indicated for patients with bone deficiency in the posterior maxilla.^1^ Various graft materials are used for sinus lift surgery, including autologous bone, xenografts, mineralized and demineralized bone allografts, and alloplasts. However, in recent years, xenografts and allografts have been used more extensively than bone autografts in sinus lift procedures, which is mostly due to a decrease in surgical complications associated with the graft donor region.^3^ Although many studies have reported success with use of xenografts and allografts, higher treatment costs and the potential of disease transmission are still notable. Thus, the possibility of sinus lift without using bone-substitute materials can be desirable.

Ellegaard et al^4^ presented the first clinical report on sinus lift procedure without any graft material. Also, Lundgren et al^5^ described open sinus lift technique without graft materials and only with clot formation.^5^ Other studies have also reported that bone formation is not dependent on the graft material, and clot formation alone can lead to formation of bone in the space created under the membrane of the sinus.^6,7^ In this method, based on GBR principles, dental implants are also inserted, so that it keeps the membrane in an elevated position as tent pegs, which can fill up the space with blood clot. The blood clot can act a scaffold for bone formation.^5^

To improve the clot stability, release more growth factors, accelerate the healing speed, improve the quality of the bone formed, and enhance bone formation, some researchers have used platelet-rich fibrin (PRF) in sinus lift surgery simultaneous with implant placement.^8-13^ PRF is prepared from venous blood with one centrifugation process. After centrifugation, three parts are formed in the test tube, with the middle layer being PRF, which is a fibrin-rich platelet gel containing a minimum level of red blood cells, while the top layer is made of plasma and the bottom layer is composed of a clot of RBCs. PRF contains coagulation factors forming a fibrin network, with different types of cytokines. PRF contains different cells, including platelets, leukocytes, macrophages, granulocytes, and neutrophils. There is no need to delay PRF formation artificially by anti-coagulants, as this even does not initiate quickly. There is no need either to increase the extent of natural blood clotting and platelet activity, as the structure of the fibrin network develops through centrifugation along with large amounts of biological factors such as entrapped cytokines.^14-16^

The present research, with split-mouth design, dealt with radiographic investigation of the height and density of bone in sinus floor elevation surgery using PRF compared with sinus floor elevation without any graft material in order to assess the quality and quantity of bone.

## Materials and Methods

### Patient selection and study design

In this split-mouth study the subjects consisted of 30‒80-year-old patients, visiting the Implant Ward of Faculty Dentistry, Tabriz University of Medical Sciences, with bilateral partial edentulism or complete edentulism, who needed sinus lift procedure plus implant placement. In the present research, one sinus on one side of each patient was randomly assigned to the PRF test group, while the other side was considered as the control group without any graft materials. The inclusion criteria were complete edentulism or posterior bilateral partial edentulism in the maxilla, with at least 4 mm and at most 8 mm of remaining bone height between the alveolar crest and the sinus floor. The exclusion criteria consisted of the presence of systemic diseases such as uncontrolled diabetes, cardiovascular diseases, malignancy, head and neck radiotherapy, and autoimmune diseases with contraindication of surgery, individuals with poor oral hygiene, smoking, and history or presence of pathological signs in the sinus.

### PRF preparation

PRF preparation was carried out similar to other studies,^8,14,15,17^ and instructions provided by Choukroun et al.^18^ Before the surgery, 20‒40 mL of the venous blood of the patient was collected in sterilized glass tubes without any anti-coagulation agent, immediately placed in the device and centrifuged at 3000 rpm for 10 minutes. Thus, activation of the coagulation cascade and formation of fibrin network of three layers, including 1) the top layer or cell-free plasma (PPP), 2) the middle layer (PRF), and 3) the bottom layer with red blood cells, were evident in the test tube. The middle layer (PRF) was separated by scissors from the clot of red globules and used as a filling material in the sinus space.

### Surgical method

The surgery was carried out under local anesthesia. Access to the sinus wall was gained in the crest of the edentulous ridge, where vertical releasing was performed at the beginning and end of the flap, and full-thickness elevation of flap was carried out. By using a diamond bur, a window was prepared in the lateral wall of the sinus. The bone window was gently separated off the membrane in order to facilitate access to the membrane. Thereafter, the membrane was slowly dragged aside off the sinus floor up to the middle wall of the sinus so that the membrane would be completely elevated. Then, preparation of the implant site was performed carefully, and 41 implants with a length of 11.5 mm (CMI IS‐II active implants, Neobiotech Co., Seoul, Korea) were placed. Next, based on the split-mouth design, 2‒4 PRFs were used on the test side in the developed space, but in the control side of the same patient, this space only filled with clot. An absorbable membrane was used on the created window and the flap was sutured on its original site. All the patients were prescribed amoxicillin (500 mg) and metronidazole (250 mg) every eight hours along with Gelofen (400 mg) every six hours up to one week. Furthermore, 0.2% chlorhexidine mouthwash was prescribed twice a day for weeks.

### Radiographic assessment

After six months, to investigate the density of the bone formed around the dental implants in the posterior maxilla, the patients underwent CBCT examinations. The bone height from the sinus floor to the crest ridge at baseline and six months after the sinus floor elevation surgery and implant placement was also measured using CBCT.

### Analysis of data

First, data normality was investigated. Kolmogorov-Smirnov test showed that the data had normal distribution (P>0.05). Thus, paired t-test was used to compare the means of indices over time (before and after surgery) and between the two groups (with and without PRF). Furthermore, paired t-test was used to compare the bone heights and densities between the test and control groups. This way the mean differences of these indices after surgery between the two groups could be attributed to the intervention adopted. The results showed that the mean measurements before the surgery were not significantly different between the two groups (P=0.706). All the tests were statistically analyzed with SPSS 21. In this study, P<0.05 was considered significant. To investigate the bone density, Hounsfield units were used with the help of Mimics 10.01 software.

### Ethical considerations

All the subjects signed informed consent forms. The protocol of the study was approved by the Ethics Committee of Tabriz Faculty of Dentistry under the code IRCT20120702010155N4.

## Results

In this split-mouth clinical trial, 14 patients with complete edentulism or bilateral partial edentulism of the upper jaw were investigated. In each case, for the sinus lift surgery of the test side, PRF was used, while in the sinus lift surgery of the other side of the same patient, no graft materials were used. Before the second-stage surgery, the subjects underwent CBCT examinations. All the 41 implants were osseointegrated in the second stage of surgery and were clinically stable. The extent of bone density formed in the two groups and the bone height prior to and after the sinus surgery were examined in the groups with and without PRF. Comparison of the bone height before and after the sinus surgery is provided in Table 1, demonstrating that the bone height in the group with PRF before the sinus surgery was 5.85±1.08 mm, which increased to 10.71±1.09 mm after surgery. The difference between the pre- and post-operative intervals in this index was 4.86 mm, which was statistically significant (P<0.001). In addition, the bone height in the group without PRF was 5.67±1.03 before surgery, which increased to 9.28±1.28 mm. The difference between pre- and post-operative intervals in this index was 3.61 mm, which was significant (P<0.001). Comparison of the mean bone heights and bone densities between the two groups is provided in Table 2. It shows that the bone height was 1.42 mm higher in the group with PRF than that in the group without PRF, which is statistically significant (P=0.004). Furthermore, the bone density in the PRF group was 52.85 units higher than that in the group without PRF, which was statistically significant (P<0.001).

**Table 1:** Comparison ofbone heights before and after sinus surgery

		**Min**	**Max**	**Mean±SD**	**Mean difference**	**P-value**
**With PRF**	Before	4.05	7.37	5.85±1.08	-4.86	<0.001
	After	8.69	12.06	10.71±1.09		
**WithoutPRF**	Before	4.11	7.18	5.67±1.03	-3.61	<0.001
	After	07.11	10.78	9.28±1.28		

**Table 2:** Comparing bone height and density in the two groups with and without PRF after sinus surgery

		Mean±SD	Mean difference between groups	P-value
**Bone height**	With PRF	10.71±1.09	-1.42	0.004
	without PRF	9.28±1.28		
**Bone density**	With PRF	310.35±40.01	-52.85	<0.001
	without PRF	257.5±35.05		

## Discussion

In recent years, bone substitute materials such as xenografts and allografts have been used more extensively than bone autografts in enhancing the sinus floor, which is mostly due to diminished surgical complications associated with the graft donor site.^3^ Furthermore, many studies have reported successful use of xenografts and allografts. However, the treatment cost is higher and the disease transfer potential is still notable. Considering the costs and risk of infection, the possibility of elevating the sinus floor without using bone substitute materials can be desirable. In the present research, the bone height and density formed in the maxilla sinus were examined using the CBCT technique after sinus membrane elevation surgery simultaneously with implant placement without any graft material or use of PRF.

In the present study the patients were followed for six months using CBCT technique. The results showed that in the sinus lift group, the bone formed beneath the membrane without any graft material and by only developing a blood clot. In the sinus lift group, without any graft material, the mean bone height from the crest to the sinus floor increased from 5.67 mm before surgery to 9.28 mm after surgery.

In a first-of-its-kind experimental study, Boyne et al^19^ reported bone formation around implants placed within the sinus up to 5 mm in monkeys. Ellegaard^4^ presented the first clinical report on bone formation around implants placed inside the sinus space up to 5 mm, concurrent with sinus lift surgery. Lundgren et al^5^ placed implants embedded into the sinus space by at least 5 mm and reported obvious formation of bone within a one-year follow-up. Chen et al,^6^ as well as Thor et al,^7^ reported that bone formation in maxillary sinus does not need the presence of biomaterials. Also, Riben et al^20^ proposed that preserving the space through implant for blood clot formation, its absorption and deposition of bone cells with periosteum origin or the maxilla spongy bone are possibly responsible for bone formation in this region. In the study by Kim et al,^21^ extraction of stem cells from Schneiderian membrane and osteogenic differentiation potential of these cells were reported. Moreover, Srouji et al^22^ showed intrinsic osteogenic potential of Schneiderian membrane and the content of osteoprogenitor cells of Schneiderian membrane,^23^ which can be possibly the origin of the new bone beneath the sinus membrane. Based on these studies, bone formation in the control group here can be justified and in line with other studies, the present research showed that if the collapse of sinus membrane is prevented by using dental implants, the formation of blood clots and periosteal osteogenic cells and the sinus floor bone may have the potential of bone formation.^7,20,24-27^

In contrast, in an animal research, Kim et al^28^ showed that when no graft material is used in sinus lift surgery, bone formation becomes limited. In line with the above study, Sul et al^29^ reported limited bone formation around implants penetrating into the sinus. Based on the present research and the studies in line with it, it may be stated that in the research by Kim et al as well as Sul et al, the limited bone formation around the implants might be explained by the membrane collapse in response to air pressure, no formation of stable clot, or not elevating the membrane properly in the animal samples.

The implants placed concurrent with sinus lift procedure can act as tent pegs. In this method, based on GBR principles, the implant is embedded concurrent with the sinus lift procedure, and no material is used beneath the space developed between the membrane and sinus floor. Indeed, the end of implants keeps the sinus membrane at an elevated position, causing the space developed to be filled by blood clot, which becomes a scaffold for bone formation, cellular migration, differentiation, and osteogenesis.^7,20,24,26,30^ Some papers have used PRF in sinus lift surgery procedures concurrent with implant placement for greater release of growth factors, increasing the healing speed, improving the quality of the bone formed, and increasing bone formation.^8-13,17,18^ In the test group of the present study, the sinus lift surgery was performed concurrent with implant placement along with use of PRF as the only graft material under the sinus membrane. In this group, the mean height of bone before the sinus surgery was 5.85 mm, which increased to 10.71 mm after surgery. As with the present research, in three studies,^11,12,31^ PRF was used as the only graft material in open sinus lift surgery concurrent with implant placement. Mazor et al^11^ performed 22 sinus lift surgeries along with implant placement. The mean initial bone height was 2.9 mm, which increased to10.1 mm in the six-month follow-up period after surgery based on radiographic evaluations, indicating a significant increase in bone height. Simonpieri et al^12^ and Tajima et al^31^ reported that the mean final height of the sinus bone, when using PRF as the only graft material in sinus lift surgery concurrent with implant placement, was 10.4 and 11.8 mm, respectively. Nevertheless, in a systematic review by Ali,^32^ it was found that these studies had no control groups in order to demonstrate the advantages of PRF as compared with sinus lift without any graft material. The present split-mouth research with a control group investigated the effect of PRF on the height and density of bone formation in sinus lift surgery. In other words, in the sinus lift surgery of the test group, PRF was used, while on the other side of the same patient as the control group, no graft material was utilized. The results of CBCT investigations of the patients six months after the surgery showed that the mean height of the sinus bone in the control group without graft material was 9,28 mm, while the mean bone height of the test group (PRF) was 10.71 mm. Therefore, the mean bone height in the group with PRF was 1.42 mm higher than that in the group without any graft material, with the difference being reported as significant (P=0.004).

Bone density can be evaluated by Hounsfield unit. Applying this parameter, a relative scale is defined which has proved valuable for different types of bone, including very high density cortical bone (>600 HU), cortical bone plus a medium density spongy bone (400 to 600 HU), and cortical bone plus low density spongy bone (<200 HU).^33^ As mentioned previously, although various studies have reported bone formation in sinus lift surgeries whether with PRF or without graft material, there are limited studies on the density of the bone formed after elevating the Schneiderian membrane.^24,31^ In the present study, the mean density of the bone formed around the dental implants of the test group (PRF) was 310.35 HU, while the mean density of the bone formed around the dental implants in the control group without graft material was 257.5 HU. Under these conditions where the bone density in the PRF group has been 52.85 units, which is significantly higher than that in the control group, in the present study, the density of the formed bone whether with PRF or without any graft material was comparable to the values reported for the normal bone present in the posterior maxillary region.^33-35^ In line with the results of the present research, Tajima et al^31^ reported that mean density of a newly formed bone six months after sinus lift surgery using PRF as the only graft material was 323 HU.^31^

Altintas et al^24^ reported bone density after sinus lift surgery concurrent with placement of dental implants in two groups without graft material and with bone allograft within one-week, three-month, and six-month follow-ups. In this research, the bone density exhibited no significant difference in the one-week and three-month follow-ups. However, in the six-month investigation, the bone density was significantly higher in the group without graft compared with the allograft group. The possible reason for this issue could be absorption process and wasting of allograft and its substitution with fresh bone, which requires 9‒12 months.^6,7^

Although the technique used for the control group in the present research did realize the goals of sinus lift surgery for embedding dental implants with a standard size, the results of the present research suggested the effectiveness of PRF. Thus, it seems that the amount of bone between the crest and the sinus floor can be a guide for using the treatment method. This means that if the extent of remaining bone is >4 mm, given the primary success of the control group, it is suggested that no graft material be used. However, if more bone is required, PRF can be used. In addition, as shown by the present research, since the bone density is higher when PRF is used, it is advisable to consider it for enhancing the bone quality in sinus lift surgeries.

## Conclusion

Considering the limitations of the study and based on the results obtained here, one can possibly say that open sinus lift surgery using PRF and even without PRF can be reliable to place implants. Nevertheless, based on the present research, PRF is suggested to enhance the quantity and quality of bone formation. It is recommended that future studies consider the extent of newly formed bone based on histological investigations.

## Authors’ contributions

The study was planned by MCh and HK. Data collection was carried out by HK; statistical analyses and interpretation of data were carried out by AHD. The manuscript was prepared by AB and SA and revised by HK. All the authors have read and approved the final manuscript for submission.

## Competing interests

The authors declare that they have no competing interests with regards to authorship and/or publications of this paper.

## Ethics approval

The study protocol was approved by the Ethics Committee in Medical Research of Tabriz University of Medical Sciences.
